# Gene expression profiling in gills of the great spider crab *Hyas araneus* in response to ocean acidification and warming

**DOI:** 10.1186/1471-2164-15-789

**Published:** 2014-09-12

**Authors:** Lars Harms, Stephan Frickenhaus, Melanie Schiffer, Felix Christopher Mark, Daniela Storch, Christoph Held, Hans-Otto Pörtner, Magnus Lucassen

**Affiliations:** Integrative Ecophysiology, Alfred Wegener Institute, Bremerhaven, Germany; Scientific Computing, Alfred Wegener Institute, Bremerhaven, Germany; Functional Ecology, Alfred Wegener Institute, Bremerhaven, Germany

**Keywords:** *Hyas araneus*, RNA-Seq, Ocean acidification, Warming, Gene expression, Crustaceans

## Abstract

**Background:**

Hypercapnia and elevated temperatures resulting from climate change may have adverse consequences for many marine organisms. While diverse physiological and ecological effects have been identified, changes in those molecular mechanisms, which shape the physiological phenotype of a species and limit its capacity to compensate, remain poorly understood. Here, we use global gene expression profiling through RNA-Sequencing to study the transcriptional responses to ocean acidification and warming in gills of the boreal spider crab *Hyas araneus* exposed medium-term (10 weeks) to intermediate (1,120 *μ*atm) and high (1,960 *μ*atm) *P*CO_2_ at different temperatures (5°C and 10°C).

**Results:**

The analyses reveal shifts in steady state gene expression from control to intermediate and from intermediate to high CO_2_ exposures. At 5°C acid–base, energy metabolism and stress response related genes were upregulated at intermediate *P*CO_2_, whereas high *P*CO_2_ induced a relative reduction in expression to levels closer to controls. A similar pattern was found at elevated temperature (10°C). There was a strong coordination between acid–base, metabolic and stress-related processes. Hemolymph parameters at intermediate *P*CO_2_ indicate enhanced capacity in acid–base compensation potentially supported by upregulation of a V-ATPase. The likely enhanced energy demand might be met by the upregulation of the electron transport system (ETS), but may lead to increased oxidative stress reflected in upregulated antioxidant defense transcripts. These mechanisms were attenuated by high *P*CO_2_, possibly as a result of limited acid–base compensation and metabolic down-regulation.

**Conclusion:**

Our findings indicate a *P*CO_2_ dependent threshold beyond which compensation by acclimation fails progressively. They also indicate a limited ability of this stenoecious crustacean to compensate for the effects of ocean acidification with and without concomitant warming.

**Electronic supplementary material:**

The online version of this article (doi:10.1186/1471-2164-15-789) contains supplementary material, which is available to authorized users.

## Background

Increasing anthropogenic emissions of CO_2_ induce ocean warming and acidification. These changes in environmental conditions may have adverse effects on marine organisms [1–5]. However, the responses to ocean acidification (OA) are highly variable between organisms [4, 5] based on the fact that various animals differ in their capacities to compensate for acid–base disturbances caused by elevated seawater CO_2_ and resulting blood hypercapnia for review see [5]. Organisms with low compensation abilities show depressed metabolism, altered energy budgets, and as a result, lower rates of growth or development [6–9]. In contrast, organisms compensating for acid–base disturbances through active ion transport, such as fish, cephalopods and some crustaceans are projected to be more tolerant towards OA [3, 10]. In parallel to these differential capacities, sensitivities within a phylum seem to be related to differences in lifestyle and associated energy turnover [5, 11]. Furthermore, species or populations from highly variable environments with natural variations in *P*CO_2_ may have evolved to be more tolerant than species from relatively stable environments. As an extreme example, the shallow living crab *Cancer magister* can compensate within 24 h for hypercapnia-induced acidosis, while the extracellular acidosis in the deep-sea crab *Chionoecetes tanneri* remains mostly uncompensated during this time [12]. However, such short-term studies have limited value if it comes to the projection of long-term ocean acidification effects.

The great spider crab *Hyas araneus* is an osmoconforming, slow-moving and inactive species living in relatively stable physical conditions and is thus an excellent candidate to study the medium to long-term effects of abiotic stressors. A number of physiological studies have already investigated the effects of elevated seawater *P*CO_2_ on this species: CO_2_ induced decreases in growth rates and fitness of larvae were demonstrated in a North Sea population, whereas an Arctic population seemed more sensitive towards thermal stress [13]. In the Spitsbergen population elevated seawater *P*CO_2_ (1,100 *μ*atm) caused an increase in metabolic rate during larval development pointing to higher metabolic costs [14]. Adult *H. araneus* became more heat intolerant under elevated CO_2_ with potential consequences for biogeographical distribution [15]. In the Arctic population synergistic effects of increased temperature and *P*CO_2_ adversely influenced the capacities for activity associated with disturbances in acid–base status [16].

To understand organismal sensitivities and tolerance-limits to OA with and without concomitant warming it is important to identify and differentiate between the mechanisms that shape an organism’s capacity to cope with the projected changes. At the whole organism level, crustaceans are impacted by OA with and without concomitant warming with effects ranging from changes in acid–base homeostasis [12, 16, 17], metabolism [9, 18, 19], growth [7, 13, 20, 21], to development [13, 14, 22, 23] and even survival [21, 24]. These processes are highly interdependent. While active acid–base regulation is an energy-consuming process [25], eventually leading to enhanced metabolic requirements [26], uncompensated extracellular pH can elicit metabolic depression [27] via effects on transmembrane ion exchange [25]. Furthermore, low pH can trigger a decrease in protein synthesis [28]; this may result in reduction of growth under hypercapnic conditions [6]. These previous studies provide us with important insights into the mechanistic background of responses to ongoing OA and warming, but also highlight the complexity of the processes involved. To elaborate the sensitivities and potential tolerance limits further, it is important to investigate the key regulatory mechanisms shaping affected processes and the tradeoffs between them.

A transcriptomic approach makes it possible to simultaneously investigate the genetic response of a wide range of cellular processes, and thus to identify the early responses to environmental changes [29]. Gene expression analyses can be used to characterize the molecular phenotype and the cellular changes that underpin physiological responses. They can also be used to uncover molecular mechanisms that might define physiological plasticity. Transcriptomic analysis can further reveal the connections between response mechanisms to environmental changes such as OA or temperature that may otherwise be overlooked [30]. Due to technological advances in recent years, analyses of the whole transcriptome have become increasingly attractive to study non-model (marine) organisms, and their molecular responses to a variety of environmental changes such as warming [31, 32], salinity fluctuations [33], hypoxia [34, 35] or OA [29, 36, 37] in marine organisms.

In the present study, we used gene expression profiling to explore the molecular response in gills of *H. araneus* exposed to hypercapnia at different temperatures. In marine crustaceans, gills are the first line of defence against acid–base disturbances of body fluids and thus the most important regulatory tissue for CO_2_ induced acidification of the hemolymph [38, 39]. We used a quantitative transcriptomic approach based on direct cDNA sequencing using high-throughput Illumina sequencing [40]. Since the present study focuses on the mechanisms involved and the potential sensitivity of *H. araneus* to climate changes, we selected CO_2_ concentrations projected for the year 2100 and 2300 by the Intergovernmental Panel on Climate Change (IPCC) as well as two different temperatures (5°C as the summer control temperature for the Arctic population and 10°C as the median habitat temperature of the species considering the whole distribution range. This study provides comprehensive insights into the transcriptional changes involved in the responses to warming and OA.

## Methods

### Animals, experimental treatments and tissue sampling

Adult specimens of the Arctic spider crab *H. araneus* (Linnaeus 1758) were collected by scientific divers in May 2009 in Kongsfjord at the west coast of Spitsbergen at 7–12 m depth (N 78°58.635'; E 11°29.454') and transferred to the Alfred Wegener Institute, Bremerhaven, Germany. Animals were maintained at 5°C in flow through aquaria with natural seawater prior to experimentation. During this period, seawater was aerated with ambient air and animals were fed *ad libitum* twice per week with frozen mussels and cockles (*Mytilus edulis* and *Cerastoderma edule*).

**Figure 1:**
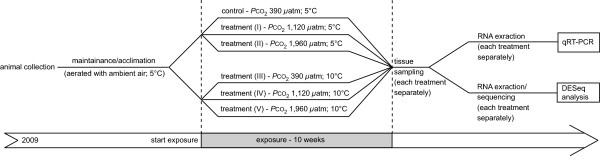
**Overview of the experimental design used in the differential expression** ***.*** Animals were collected in 2009 and acclimated to 5°C until the start of experimentation. Subsequently, exposure experiments were conducted for a time period of 10 weeks for all treatments. After exposure, tissue samples were taken and total RNA was extracted for analyses by quantitative real-time polymerase chain reaction (qRT-PCR) and Sequencing. Sequencing data were used for differential expression analysis by DESeq.

For the medium-term experiment, male spider crabs with a carapace width of 26 to 42 mm were divided into six groups and each group was randomly assigned to the different treatments. Animals were exposed to three different CO_2_ concentrations (390 *μ*atm as control, 1,120 *μ*atm as intermediate concentration and 1,960 *μ*atm as high CO_2_ treatment) and two different temperatures (5°C as control and 10°C as elevated temperature) for 10 weeks (Figure 1). For each treatment, 5–7 animals were individually placed in 2 l wide-mouth containers (Kautex, Bonn, Germany).

Experiments were carried out in recirculating seawater CO_2_ manipulation systems of 1 cubic meter volume each. Seawater of the storage tank was pumped to a header tank at a rate of 20 l min^−1^, which supplied the wide-mouth container by gravity feed at a flow rate of 200 ml min^−1^. Water of experimental containers was retained in a collection tank and pumped back to the storage tank at a flow rate of 20 l min^−1^. Ambient air temperature in the experimental rooms was thermostated to keep water temperature constant. Seawater CO_2_ manipulation was accomplished by constantly aerating the storage and header tanks with a defined air/CO_2_ mixture using an automatic mass flow controller (HTK 6 channel, HTK Hamburg GmbH, Germany). A light–dark cycle of 12:12 h was established. Water was partly changed every week by refilling the storage tank with pre-equilibrated seawater (*P*CO_2_ and temperature). Experimental animals were fed once a week *ad libitum* with frozen mussels (*C. edule*).

To monitor water physicochemistry, seawater samples were collected in airtight glass vials to prevent exchange with the atmosphere, and total dissolved carbon (DIC) concentration was immediately measured with a Seal QuAAtro SFA Analyzer (Seal Analytical, Mequon, United States of America). Temperature, salinity and pH were measured at the time of collection and, together with DIC, used to calculate the *P*CO_2_ in seawater using CO2SYS [41]. Seawater pH was measured using a pH electrode (ProfiLine pH 3310, WTW Wissenschaftlich-Technische Werkstätten GmbH, Weilheim, Germany) calibrated at the respective temperature with National Institute of Standards and Technology (NIST) standard pH buffer and salinity with a conductivity meter (ProfiLine Cond 1970i, WTW Wissenschaftlich-Technische Werkstätten GmbH, Weilheim, Germany). For CO2SYS, NBS (NIST) scale of seawater pH and constants of Mehrbach *et al*. [41] refitted by Dickson and Millero [41] were used. A summary of water physicochemistry data is given in Table 1.

**Table 1:** **Summary of the seawater physiochemical conditions during experiments with** ***Hyas araneus***

Parameter	Control	Treatment (I)	Treatment (II)	Treatment (III)	Treatment (IV)	Treatment (V)
Temperature (°C)	5.3 ± 0.2	4.2 ± 0.2	4.5 ± 0.2	9.9 ± 0.2	9.7 ± 0.3	9.8 ± 0.2
Salinity (‰)	32.1 ± 0.7	32.2 ± 0.7	32.2 ± 0.6	33.6 ± 0.4	33.5 ± 0.4	33.6 ± 0.3
pH (NBS scale)	8.15 ± 0.03	7.81 ± 0.04	7.55 ± 0.06	8.22 ± 0.04	7.85 ± 0.04	7.54 ± 0.05
DIC (mmol kg^−1^)	2,366 ± 42	2,436 ± 14	2,520 ± 39	2,295 ± 28	2,395 ± 14	2,488 ± 22
*P*CO_2_ (*μ*atm)	441 ± 35	991 ± 96	1,878 ± 246	366 ± 30	942 ± 59	2,015 ± 147
Total alkalinity (mmol kg^−1^)	2,479 ± 13	2,479 ± 14	2,491 ± 14	2,484 ± 10	2,469 ± 13	2,473 ± 15

Temperature, Salinity, pH and dissolved inorganic carbon (DIC) were measured and partial pressure of CO_2_ (*P*CO_2_) and total alkalinity were calculated using CO2SYS [41]. Data are mean ± SD with N = 24 (5°C), N = 16–20 (10°C).

After experimental exposure, all 6 gill pairs were collected from 5–7 animals in each treatment. Tissue samples were immediately frozen in liquid nitrogen and stored at −80°C until usage.

### Hemolymph sampling and measurements

Directly before tissue sampling, around 1 ml of hemolymph was extracted at the coxa of the third walking leg using a 1 ml sterile syringe (Henke-Sass, Wolf GmbH, Tuttlingen, Germany). Hemolymph was immediately transferred to a 1.5 ml tube (AG Eppendorf, Hamburg, Germany), placed in a thermostatted water bath and pH was measured at acclimation temperature using a pH microelectrode (PHM 93 Reference pH meter, Radiometer, Copenhagen, Denmark; InLab Micro, Mettler Toledo GmbH, Germany). The pH meter was calibrated at the respective temperature with NIST standard pH buffer. A hemolymph subsample was withdrawn using a gas-tight 200 *μ*l syringe (Hamilton Company, Reno, United States of America) and total dissolved inorganic carbon (CCO_2_) of extracellular fluid was analysed according to the modified gas chromatographic method [42, 43]. Extracellular fluid was injected in gas tight glass vials containing 3 ml of air equilibrated 0.1 M hydrogen chloride (HCl) and analysed by gas chromatography in an Agilent 6890 N GC System (Agilent Technologies, Santa Clara, United States of America). The bicarbonate (HCO_3_^−^) concentration of the extracellular fluid was calculated from CCO_2_ and pH using equations derived from the Henderson-Hasselbalch equation. *P*CO_2_ was calculated as *P*CO_2_ = *C*CO_2_ * (10^ph-pkIII^ * αCO_2_ + αCO_2_)^−^1 and HCO_3_^−^ as HCO_3_^−^ = *C*CO_2_- αCO_2_ * *P*CO_2_, with *C*CO_2_ being the total CO_2_ concentration in mM, αCO_2_ the physical solubility of CO_2_, *P*CO_2_ the partial pressure of CO_2_ in kPa and pK the apparent dissociation constant of the CO_2_/apparent HCO_3_^−^ system. αCO_2_ and pK were calculated according to Pörtner et al. [44]. Raw data of hemolymph sampling and measurements are available at http://doi.org/10.1594/PANGAEA.833705.

### RNA extraction and sequencing

Total tissue RNA of gills was extracted using the RNeasy Mini Kit according to the Purification of Total RNA from Animal Tissue protocol (QIAGEN, Hilden, Germany). RNA quantities were determined by a NanoDrop 2000c spectrometer (PeqLab, Erlangen, Germany), and RNA was analysed for quality by microfluidic electrophoresis in an Agilent 2100 Bioanalyzer (Agilent Technologies, Santa Clara, United States of America). Total RNA from all gill pairs of 4 animals was pooled for each treatment, and used for library constructions and sequencing by GATC Biotech (Konstanz, Germany). The cDNA libraries for each treatment were constructed according to the SMART protocol for Illumina sequencing (Clontech, Mountain View, USA) and after adapter ligation pooled into two samples. To obtain appropriate deep sequencing results, samples were sequenced at least twice. Illumina sequencing was performed on a HiSeq 2000 Sequencer by GATC Biotech (Konstanz, Germany). Raw reads were quality controlled by FastQC (Babraham Institute, Cambridge, UK) and cleaned using the FastX-Toolkit (Hannon Lab - Cold Spring Harbor Laboratory, New York, USA). Quality control and trimming was performed using the following parameters: Minimum quality score of 20, minimum percentage of bases within the quality score of 90 and a minimum length of 25 bases. The cleaned raw data of the Illumina sequencing were deposited in the European Nucleotide Archive (ENA) at the European Molecular Biological Laboratory – European Bioinformatics Institute (EMBL-EBI) (http://www.ebi.ac.uk/ena/data/view/ERP002128). A summary of the cleaned sequencing results for all samples is given in Additional file 1: Table S1.

### Mapping and identification of differentially expressed genes

Short reads of each sample were separately aligned against the annotated *H. araneus* transcriptome [40], using the Burrows-Wheeler Aligner (BWA) (version 0.5.9) with default parameters [45]. Obtained files were processed into bam files for further analysis, using SAMTools (version 0.1.18) [46]. An overview of the mapping and efficiency is described in Additional file 1: Table S1. Differential expression analysis was conducted with the R statistic software [47]. Read counts were summed up for all sequencing runs of each sample and used for the differential expression analysis without biological replicates. Differential expression of genes was evaluated using a test based on the negative binomial distribution as integrated in the Bioconductor R package DESeq [48], with a standard level of *p* ≤ 0.05 indicating significance. Control (control *P*CO_2_/control temperature) was compared to five treatments: (I) elevated temperature; (II) intermediate *P*CO_2_ at control temperature; (III) high *P*CO_2_ at control temperature (IV) intermediate *P*CO_2_ at elevated temperature; (V) high *P*CO_2_ at elevated temperature. The previously annotated transcriptome made a Gene Ontology enrichment analysis possible to test for particular affected terms, using Fisher’s exact test (FDR ≤ 0.05) as implemented in the Blast2GO software (version 2.6.0) [49, 50]. All subsets of significantly regulated genes identified by the binominal distribution test were tested against the full set of annotated sequences of the *H. araneus* transcriptome. To cut down on redundancy, GO terms were summarized into a more representative subset of terms using the web-based clustering tool REVIGO [51].

### Validation by quantitative real-time polymerase chain reaction (qRT-PCR)

A set of transcript sequences known to be involved in acid–base regulation and/or transcripts that showed differential expression in one or more treatments was selected for validation of RNA-Seq results. Primers were designed using the PrimerExpress software (version 3.0) (Applied Biosystems, Darmstadt, Germany) with the Taq-Man MGB Quantification method and default parameters (Additional file 2: Table S2). Primer specificity was given by using sequences of the annotated *H. araneus* transcriptome [40]. All primer pairs were tested for performance and efficiency across a series of cDNA dilutions (1:20; 1:40; 1: 100; 1:200; 1:1000; 1:2000). Primers used displayed a suitable per cycle amplification rate, with an efficiency (*E*) of 2.0 ± 0.1 and R^2^ > 0.98. Efficiency was calculated as *E* = 10^(−1/S)^, with s being the slope of linear regression.

Total RNA was extracted from gills as described above. Ten micrograms of total RNA per sample was treated with DNAse for DNA digestion using the Turbo DNA-free kit (Ambion, Darmstadt, Germany) and 0.4 *μ*g DNA free RNA was transcribed into cDNA with the High-Capacity cDNA Reverse Transcription kit (Applied Biosystems, Darmstadt, Germany). Real-time PCR was performed on a 7500 Real-time PCR System (Applied Biosystems, Darmstadt, Germany) and SYBR® Green PCR master mix (Applied Biosystems, Darmstadt, Germany). All genes were finally analysed in a 40-fold dilution and amplified with 300 nM of primer. To verify the amplification specificity of fragments a melting curve analysis was performed for each reaction.

Gene expression calculation was based on the C_T_-threshold. Absolute mRNA quantities were calculated as *Q*_X_ = *E*^(CT)^ and normalized with the formula *Q*_N(X)_ = *Q*_X_/*Q*_X(HK)_, with *Q*_X(HK)_ being the absolute mRNA quantity of the housekeeping gene sodium bicarbonate cotransporter (NBC). The housekeeping gene was determined using geNorm implemented in the software qbasePlus (version 2.1) (Biogazelle, Zwijnaarde, Belgium) with a relative expression stability of *M* ≤ 0.42 (high reference stability is given at an average geNorm of *M* ≤ 0.5). To ensure consistency with the differential expression results of the DESeq analysis, gene expression results of the qRT-PCR were calculated as log_2_ fold change (log_2_FC) of mean normalized quantities of treatment and control.

### Statistics

To identify significant differences in the sum of all significantly up- and down-regulated transcripts between treatments, significantly changed transcripts in one or more treatment, identified by the DESeq analysis, were transformed into a matrix with 1 = significantly up-regulated, −1 = significantly down-regulated and 0 = not significantly regulated transcript. Treatments were analysed for statistical differences applying the Wilcoxon matched pairs test as implemented in SigmaPlot (Version 12.0, Systat Software Inc., San Jose, USA) with *p* < 0.05. Data from each treatment were tested against each other.

The correlation between the differential expression results of the DESeq analysis and the corresponding gene expression results of the qRT-PCR was determined by Pearson Correlation as implemented in SigmaPlot 12.0 (Systat Software Inc., San Jose, USA).

A one-way ANOVA was used to identify the effect of seawater *P*CO_2_ on hemolymph pH and bicarbonate (HCO_3_^−^). Data obtained under various *P*CO_2_ levels were tested against each other for each temperature separately. A Holm Sidak test for multiple comparisons was used for *a posteriori* analyses. Tests were performed in SigmaPlot (Version 12.0, Systat Software Inc., San Jose, United States of America) with *p* < 0.05 indicating significant differences.

## Results and discussion

A total of 55 million reads (56%) from initial Illumina sequencing passed the quality filter and was used for the differential expression analysis. After processing, an average of 9.2 million high quality reads were produced for each sample from 2–3 sequencing runs per sample (Additional file 1: Table S1). To obtain the differential expression of each gene, high quality reads were aligned on the *H. araneus* transcriptome [40]. An average of 5.2 million reads for each sample produced distinct alignments. The alignment process yielded an average efficiency of 56% for the high quality reads (Additional file 1: Table S1). The achieved mapping efficiency is actually higher than in a comparable study of a non-model organism, which used an analogous approach for differential expression analysis (41% efficiency) [52]. Furthermore, 96.5% of all transcript sequences in the *H. araneus* transcriptome were detected in the RNA-Seq data. However, the occurrence of a large amount of unmapped reads might result from sequencing errors, repetitive sequences or inadequate quality filtering of the Illumina reads. Furthermore, it has to be considered that transcripts supported by only a small number of aligned reads (≤10) may reflect incompletely assembled transcripts in the reference. Those poorly supported transcript sequences were excluded from the subsequent analysis, and a final test-set of 16,201 transcripts sequences was used for the differential expression analysis. As there were no biological replicates, it has to be considered that the variance for genes can only be estimated by comparing the mean-variance relationship between samples/treatments, as if they were replicates, resulting in an overestimation of the variance and thus make this approach more conservative. Furthermore, expression levels of stable and highly expressed genes, based on the RNA-Seq data, were analysed by quantitative real-time PCR (qRT-PCR) confirming the RNA-Seq methodology used in this study (Additional file 3: Figure S1 and Additional file 4: Figure S2).

**Figure 2:**
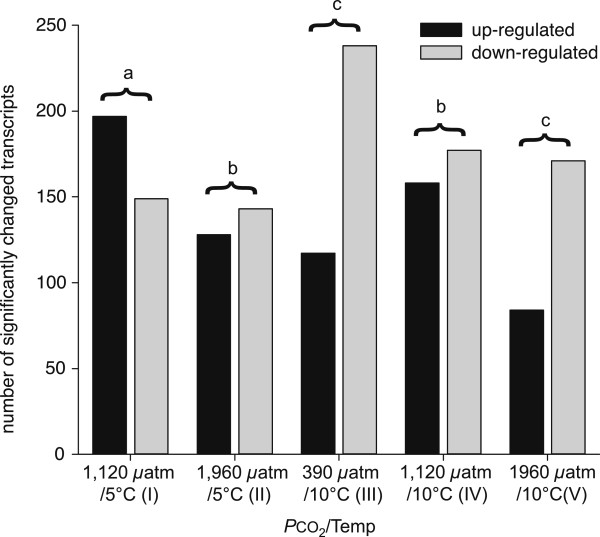
**Differences in the sum of all significantly regulated transcripts of** ***Hyas araneus*** **after exposure experiments.** For each treatment, up- and down-regulated transcripts were counted and represented as bars (black bars = up-regulated; grey bars = down-regulated). Treatments were tested for statistical differences using the Wilcoxon signed rank test (*p* < 0.05). Letters denote the significant differences. Differing letters indicate significant differences; identical letters indicate no significant difference.

We could identify 864 (5.3%) out of the 16,202 tested transcripts to be differentially expressed after medium-term (10 weeks) exposure to the abiotic effectors (Additional file 5: Table S3). Out of these differentially expressed genes (DEG) 40.0% and 31.3% were differentially expressed under intermediate CO_2_ (treatment I) and high CO_2_ (treatment II; Additional file 6: Figure S3A, B), respectively. For the high temperature (treatment III), 41.0% showed significantly different expression levels (Additional file 6: Figure S3C). The combination of factors intermediate CO_2_ (treatment IV) and high CO_2_ (treatment V) with elevated temperature revealed 38.7% and 29.4% DEG, respectively (Additional file 6: Figure S3D, E). While the total amount of significant DEGs was similar in all treatments, individual genes displayed large differences in up- or down-regulation (Figure 2). Intermediate CO_2_ (I) led to strongly up-regulated transcript levels, while high temperature (III) caused strong down-regulation.

These general results, especially the strong up-regulation of gene expression under intermediate CO_2_ (I), suggest that the regulatory capacity is high at moderately elevated *P*CO_2_ in adult *H. araneus* but reduced at higher CO_2_ levels. Up-regulation was also reduced at intermediate CO_2_ when combined with elevated temperature (IV) rather than at control temperature. Down-regulation in DEG predominated under warming alone (treatment III, high temperature) when compared to control temperature (I). 137 of the 177 genes that displayed significantly changed transcripts in the intermediate CO_2_/elevated temperature treatment (IV) were also differentially expressed in the high temperature treatment (III) supporting a strong temperature-dependent response, in line with the hypothesis that temperature affects most biochemical processes. Faced with a long-term temperature change, ectothermal organisms retain physiological homeostasis by several acclimation strategies, which can be of quantitative, qualitative or modifying nature [53]. The enhanced down-regulation seen in the high temperature treatments suggest that *H. araneus* may adopt a quantitative strategy to maintain physiological rates by down-regulating the concentrations of enzymes between 5 and 10°C [54].

To identify processes actually responding to elevated *P*CO_2_ and temperature, a first analysis was carried out using gene ontology (GO) terms. The set of GO-annotated differentially expressed genes was statistically tested for the over- and underrepresentation of GO terms to identify molecular functions, cellular components and biological processes affected most by the experimental treatments (Additional file 7: Table S4). The GO enrichment analysis revealed a variety of significantly over-represented GO terms that can primarily be summarized underneath the more generic categories ‘metabolism’ and ‘cell structure’. 23 and 25 over-represented GO terms, respectively, could be associated with these categories. It is important to mention that both intermediate CO_2_ treatments (I, IV) constitute 57% and 27% of the over-represented GO terms, respectively, representing compensation mechanisms mentioned above. The only over-represented GO term under high CO_2_, *trehalose metabolic process*, was evaluated for the down-regulated genes of the combined stress treatment (V). The fact that it was over-represented in all treatments indicates that enhanced expression of trehalose metabolism can be rated as a unifying response to both elevated CO_2_ and elevated temperature.

Within the *‘cell structure’* related GO-terms, intermediate CO_2_ (I, IV) led to significant up-regulation of genes concerning *cell surface*, *extracellular matrix*, *structural molecule activity* or *brush border membrane*, suggesting a structural modification of the gills. Some GO terms were found underrepresented at high temperature and in combined, intermediate CO_2_ and high temperature treatments (III, IV), and are assigned mainly to intracellular structures such as organelles. The over-representation of *‘cell structure’* related GO terms suggests that gill epithelial structure is adjusted in response to *P*CO_2_ disturbances. Gills are the principal organs for gas exchange and, together with the excretory organs, responsible for osmotic and ionic regulation in crustaceans [55]. As passive ion transport is influenced by the conductivity of gill epithelia [56], their structural modification might lead to a change in conductivity and would change the diffusion rate of ions. Structural changes were in fact identified in gills of *Carcinus maenas* during salinity exposure, with a modification of the apical plasma membrane system and an enlargement of the subcuticular compartment [57]. As environmental hypercapnia and salinity changes can cause similar mechanistic responses [39], similar transcriptomic modifications may occur. This is supported by an over-representation of the GO term *response to salt stress* in the intermediate CO_2_ treatment (I).

Although a GO enrichment analysis offers initial insights into processes affected by hypercapnia exposure and elevated temperature, a strong bias exists towards conserved and well-characterized processes, functions and cellular components in model organisms. This bias particularly applies to *H. araneus*, with a lack of GO annotation for about 76% of the transcripts. Additionally, many genes are grouped into more than one GO term depending on their resolution and are thus difficult to interpret. In light of these contraints, GO analysis can only provide a general overview of possibly affected processes and make a more detailed look indispensible.

For a more comprehensive understanding of the mechanisms responding to *P*CO_2_ and temperature changes, we performed a second analysis. Here, all genes included in the most affected categories *‘metabolism’* and *‘structural modification’*, identified by the GO enrichment analysis, were considered. Additionally, we integrated all genes related to *‘acid–base and ion regulation’* and *‘response to stress’* into our analysis, as adjustments in these mechanisms are likely relevant in shaping resistance to hypercapnia exposure or heat stress.

For the interpretation of transcriptomic results, it has to be considered that expression profiles represent one regulatory level in the response to environmental changes and thus do not necessarily reflect the changes of other regulatory levels, e.g. protein abundances and activities. At least 25% of the proteome cannot be covered by gene expression profiling [30]. However, there was a positive correlation between transcription and translation in 87% of genes that changed ≥ twofold in living cells of yeast strains [58]. Certainly, a lower correlation has to be considered for more complex organisms, however an additional proteomics study on *H. araneus* revealed a comparable correlation between gene expression and protein abundance of ~70% for the intermediate treatment at 5°C (unpublished). A transcriptomic approach can provide first insights into the regulatory processes responding to environmental changes such as OA or temperature.

### Response of specific groups

#### Acid–base regulation

**Figure 3:**
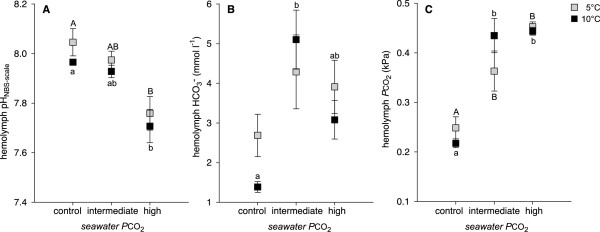
**Hemolymph acid–base status** ***Hyas araneus*** **(A, pH-values; B, bicarbonate levels; C,** ***P*** **CO**_**2**_ **levels) in response to different exposure experiments.** Squares represent the means with error bars depicting the standard error for each treatment. Grey squares refer to treatments at 5°C and black squares to treatments at 10°C. One-way ANOVAs were used to identify the effect of seawater *P*CO_2_ concentration on hemolymph pH, bicarbonate (HCO_3_^−^) and *P*CO_2_. A Holm-Sidak test for multiple comparisons was used for *posteriori* analysis (*p* < 0.05). Differing letters indicate significant differences; identical letters indicate no significant difference. Capital letters denote differences for 5°C treatments and lower cases for 10°C treatments.

**Figure 4:**
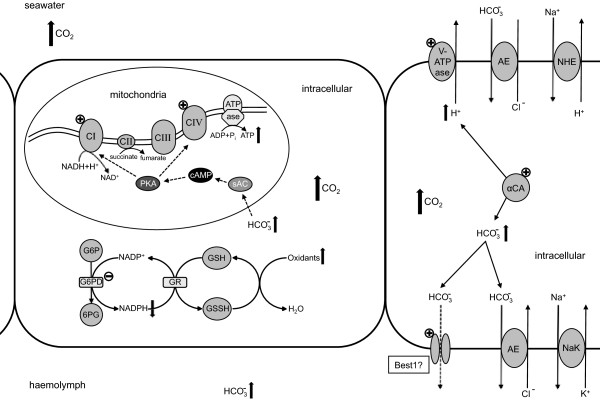
**Schematic description of proposed processes in the gill epithelium of** ***Hyas araneus*** **in response intermediate** ***P*** **CO**_**2**_ **exposure.** Medium-term hypercapnia acclimation leads to a shift to a new acid–base equilibrium by accumulation of hemolymph bicarbonate (HCO_3_^−^). CO_2_ is hydrated into H^+^ and HCO_3_^−^ by cytoplasmic carbonic anhydrase (CA). Protons are actively pumped out of the epithelial cell by an apical vacuolar proton ATPase (V(H^+^)-ATPase), followed by a transport of HCO_3_^−^ via a basolateral anion exchanger (AE) and/or ion channel, such as bestrophin (Best1). Increased energy demand is in part met by an enhanced expression of complex I (CI) and complex IV (CIV) of the electron transport system and possibly triggered by a soluble adenylyl cyclase (sAC) induced signalling pathway. sAC is stimulated by HCO_3_^−^ and increases the formation of cyclic adenosine monophosphate (cAMP), which activates protein kinase A (PKA) that subsequently leads to an induced expression of CI and CIV. While enhanced aerobic metabolic processes increase the generation of oxidants, the cellular defence against oxidative stress is adversely affected by a lower production of NADPH due to a decrease of the pentose phosphate pathway enzyme glucose-6-phosphate dehydrogenase (G6PD). NADPH acts as reducing agent for the regeneration of reduced glutathione (GSH) to oxidised glutathione (GSSH). The symbols (+) and (−) mark significantly up- and down-regulated genes, respectively modified after [55, 64].

The extracellular pH (pH_e_) measured in the hemolymph of adult *H. araneus* showed partial compensation under intermediate CO_2_ levels (I, IV) involving an increase in bicarbonate (HCO_3_^−^) concentration (Figure 3A,B). Under high CO_2_ (II, V), reduction in pH_e_ was greater and the increase in  was reduced (Figure 3A,B). These findings suggest a limited capability to compensate for pH_e_ disturbances caused by high seawater *P*CO_2_. According to a crustacean model by Freire et al. [55], proton (H^+^) excretion is generated by apical vacuolar-type (H^+^)-ATPase (V(H^+^)-ATPase) and/or sodium/proton exchanger (NHE), the latter dependent on sodium/potassium-ATPase (Na^+^/K^+^-ATPase). HCO_3_^−^ is enriched in the hemolymph by basolateral anion exchangers. Enzymes that support active ion transport are the intracellular carbonic anhydrase (CA) and, in terms of a general support of energy consuming mechanisms, arginine kinase (AK). CA is assumed to accelerate the dissociation of carbonic acid (H_2_CO_3_) and provide the substrate for H^+^ and HCO_3_^−^ transporters [55]. AK catalyses the reversible dephosphorylation of phosphoarginine, contributing to the restoration of adenosine triphosphate (ATP) used in energy consuming processes [59]. The expression of corresponding genes, V(H^+^)-ATPase, AK and partial sequences of two alpha CAs was significantly up-regulated at intermediate CO_2_, whereas such mRNA concentrations were only moderately increased at high CO_2_ (II) (Table 2). These expression levels follow the course of the hemolymph HCO_3_^−^ parameters and the more effective acid–base regulation of adult *H. araneus* under moderately elevated CO_2_. However, only a few sequences encoding for Na^+^/K^+^-ATPase were up-regulated under CO_2_, in contrast to their response to elevated temperature treatment (III). Under the combined effect of temperature and CO_2_ Na^+^/K^+^-ATPase was also up-regulated. Another enzyme, DOPA decarboxylase, which catalyses the biosynthesis of dopamine by decarboxylation of L-DOPA, was found down-regulated at moderate (intermediate) CO_2_ elevations (I). Elevated dopamine leads to increased sodium influx and concomitantly, increased Na^+^/K^+^-ATPase activity in gills of *C. maenas* [60]. In light of this finding constant mRNA levels of Na^+^/K^+^-ATPase at intermediate CO_2_ (I) indicate that the down-regulation of DOPA decarboxylase may even prevent an activation of Na^+^/K^+^-ATPase (Table 2). The gene expression of other transporters, supposed to be involved in acid–base regulation, such as NHE and/or bicarbonate/chloride co-transporter were not influenced at all by elevated *P*CO_2_ values or temperature. Acid–base regulation predominantly via the V(H^+^)-ATPase (as seen in the present transcriptome) might involve minimal disturbance to ionic composition, e.g. cellular sodium homeostasis which would be affected by strong involvement of Na^+^/K^+^-ATPase. In the sipunculid *S. nudus*, extracellular acidosis induced a shift in ion transporters during hypercapnia from high to low energy cost mechanisms of acid–base regulation, resulting in decreased Na^+/^K^+^-ATPase activity due to lower requirement for sodium regulation [25, 27]. In line with our present observations these results suggest a pH regulation system independent of Na^+^/K^+^-ATPase being used under hypercapnic exposure (Figure 4). No significant up-regulation of apical bicarbonate anion exchangers was observed. In *C. sapidus*, CO_2_ induced acid–base disturbances (*P*CO_2_ 10,000 μatm) were mainly compensated by an uptake of HCO_3_^−^ from the surrounding seawater via the gill epithelia [39]. However, our results with no up-regulation of respective transporters suggests hemolymph buffering may be achieved through the dissociation of respiratory CO_2_ resulting in an accumulation of HCO_3_^−^ in the hemolymph. This might be exceptionally for crustaceans with low compensatory capacities. In *H. araneus*, also no significant up-regulation of basolateral bicarbonate anion exchangers was observed. However, expression of the anion channel bestrophin was significantly enhanced (Table 2, Figure 4). Bestrophin is activated by calcium (Ca^2+^) and enhances membrane permeability for anions, such as chloride and HCO_3_^−^ [61]. Even though bestrophin is commonly found in the retinal pigment epithelium, ion channels of this family have been identified in different tissues and are involved in a variety of cellular processes [62]. Its role in responses to elevated CO_2_ remains to be investigated.

**Table 2:** **Regulation of transcripts of specific interest in the hypercapnia and elevated temperature experiments on** ***Hyas araneus***

Accession no.	Description	Rank	Treatment (I)	Treatment (II)	Treatment (III)	Treatment (IV)	Treatment (V)
HAAI01016321	uricase	10	***−5.24***	***−4.27***	***−3.68***	***−7.10***	***−6.20***
HAAI01006676	trehalose-6-phosphate synthase 1a	21	***−4.45***	***−2.42***	***−2.13***	***−6.05***	***−4.57***
HAAI01003297	cuticle proprotein	24	**5.88**	1.22	2.81	3.98	1.87
HAAI01001762	actin	53	−1.65	0.48	**5.06**	−0.34	−1.03
HAAI01004150	trehalose 6-phosphate synthase 1	61	***−4.35***	−1.68	***−2.73***	***−4.88***	***−3.99***
HAAI01015640	vitellogenin like	66	**4.82**	3.35	0.68	**3.28**	−0.13
HAAI01018061	peroxiredoxin	79	**4.67**	4.31	1.81	3.92	4.09
HAAI01010911	enoyl COA hydratase	80	−1.11	−0.65	0.34	−0.47	***−4.67***
HAAI01016834	like adducin related protein	83	**4.62**	3.22	0.81	3.30	0.29
HAAI01016838	GSH peroxidase like	87	3.67	**4.61**	2.81	3.92	4.19
HAAI01015788	vitellogenin like	100	**4.48**	3.61	1.22	3.60	−0.71
HAAI01000380	glucose-6-phosphat dehydrogenase	105	***−3.16***	***−4.45***	***−2.56***	***−3.41***	***−2.87***
HAAI01017747	sodium glucose cotransporter	113	2.94	**4.36**	0.64	1.98	1.29
HAAI01002706	trehalose 6-phosphate synthase 1b	117	***−3.87***	−1.10	***−2.20***	***−4.32***	***−3.11***
HAAI01012389	isocitrate dehydrogenase I	128	−1.18	−2.36	−0.55	***−4.19***	−2.88
HAAI01000602	dopa decarboxylase	138	***−3.37***	***−2.78***	***−2.26***	***−2.80***	***−4.09***
HAAI01003033	troponin I	175	−0.33	1.22	3.78	−0.02	0.29
HAAI01015542	heat shock protein 90	192	***−3.07***	1.07	***−3.68***	−0.09	−3.30
HAAI01002164	alpha carbonic anhydrase	197	**3.67**	1.80	1.64	**3.45**	1.87
HAAI01019079	ascorbate peroxidase	198	**3.67**	2.11	1.17	2.23	−2.52
HAAI01009105	heat shock protein 90	203	**3.61**	2.42	0.36	1.98	−2.03
HAAI01019113	vitellogenin like	226	**3.52**	**2.39**	−0.36	**2.48**	−1.56
HAAI01018669	vitellogenin like	234	**3.50**	2.29	0.15	2.00	−2.03
HAAI01016527	cytochrome p450 like	236	**3.23**	2.03	0.07	**3.47**	2.61
HAAI01009026	cuticle protein like	291	−0.47	**3.20**	0.01	0.98	−0.36
HAAI01010727	gelsolin precursor	303	1.67	0.68	**3.13**	0.64	1.29
HAAI01018844	alpha tubulin	309	**3.11**	1.41	1.46	**2.74**	−0.20
HAAI01008700	arginine kinase	338	**3.01**	2.27	1.43	**2.76**	2.14
HAAI01019135	carbohydrate phosphorylase like	344	**2.67**	1.48	1.26	**2.99**	1.21
HAAI01004058	cuticle protein	359	**2.93**	0.22	−0.55	0.20	−0.71
HAAI01015598	heat shock protein 90	367	**2.90**	1.47	−0.19	1.85	−0.71
HAAI01019120	bestrophin like	368	**2.89**	1.8	2.13	1.56	1.16
HAAI01000761	heat shock protein 70	381	**2.84**	**1.94**	1.30	**2.61**	1.37
HAAI01005842	cuticle protein like	386	**2.81**	0.48	1.54	0.63	−0.45
HAAI01001265	beta tubulin	403	**2.77**	**1.77**	0.97	**1.98**	1.02
HAAI01018645	V1-ATPase subunit	427	**2.69**	1.80	1.27	**2.54**	1.46
HAAI01018783	cuticle protein	435	**2.67**	0.71	1.49	1.50	1.61
HAAI01004930	heat shock protein 90	447	**2.63**	1.86	1.24	**2.58**	0.92
HAAI01007246	vitellogenin like	455	**2.61**	2.15	−1.10	0.98	−1.45
HAAI01003327	actin	461	**2.53**	**2.26**	1.06	**2.60**	1.72
HAAI01000796	alpha tubulin	466	**2.59**	**2.06**	1.09	**2.34**	1.70
HAAI01018213	alpha-glucosidase	477	1.83	1.26	1.56	**2.55**	2.39
HAAI01005237	alpha carbonic anhydrase like	478	2.55	1.80	0.52	**2.42**	0.91
HAAI01001455	cuticle protein like	498	−1.26	***−1.86***	−0.58	***−2.44***	***−2.51***
HAAI01014269	cuticle protein like	514	−0.50	−1.08	−0.57	−1.66	***−2.48***
HAAI01008420	troponin I	519	0.02	−0.73	***−2.46***	−0.88	−1.81
HAAI01002591	superoxide dismutase	535	−0.10	***−1.65***	−0.62	−1.49	***−2.42***
HAAI01019124	na + k + −atpase alpha subunit	536	0.81	0.22	1.82	**2.09**	2.41
HAAI01007529	glyceraldehyde 3-phosphate dehydrogenase	546	2.39	1.60	0.69	**2.36**	1.07
HAAI01004651	actin	556	2.38	1.44	0.52	**1.82**	1.33
HAAI01005807	cytochrome c oxidase subunit ii	559	**2.23**	**1.82**	0.96	**2.37**	1.33
HAAI01001460	cuticle protein like	560	−0.72	−1.31	−0.72	***−1.62***	***−2.37***
HAAI01001217	cuticle protein	562	**2.37**	1.10	**2.21**	0.40	−0.98
HAAI01003904	ankyrin related protein like	576	1.33	1.27	**2.33**	0.82	0.59
HAAI01000424	cytochrome c oxidase subunit i	583	**2.32**	**1.56**	1.10	**2.28**	0.84
HAAI01001438	nadh dehydrogenase subunit	598	**1.95**	1.46	0.87	**2.27**	1.29
HAAI01006730	heat shock protein 90	608	**2.24**	1.44	0.96	**2.13**	1.29
HAAI01005819	cuticle protein like	614	1.55	**1.87**	0.79	**2.14**	**2.23**
HAAI01015787	alpha tubulin	630	2.20	0.85	0.47	**1.88**	−0.27
HAAI01002070	vitellogenin like	634	2.20	1.77	−1.05	1.32	−1.57
HAAI01014788	thioredoxin	647	−1.73	***−2.18***	−0.50	−1.38	−1.52
HAAI01006091	gelsolin precursor	670	0.84	0.73	**2.10**	0.37	−0.28
HAAI01000874	alpha tubulin	673	**2.03**	**2.09**	0.90	1.31	1.69
HAAI01000485	cytochrome c oxidase subunit iii	711	**1.94**	1.28	0.60	**2.02**	0.90
HAAI01008219	gelsolin precursor	738	0.89	0.76	**1.97**	0.25	−0.35
HAAI01014184	thioredoxin	751	−1.08	−0.93	−1.23	***−1.94***	−1.80
HAAI01002593	thioredoxin peroxidase	769	***−1.89***	−0.72	−0.98	−1.52	−1.52
HAAI01005614	cuticle protein like	804	1.44	0.78	**1.79**	1.18	−0.21
HAAI01006347	cuticle protein like	817	1.28	−0.22	**1.75**	1.37	1.42
HAAI01018927	spectrin like	822	1.58	0.51	**1.74**	1.09	0.50
HAAI01000352	nesprin like	827	**1.70**	0.72	**1.73**	1.42	1.58

Transcripts significantly regulated in response to hypercapnia and elevated temperature as identified by DESeq analysis (for details, see Methods). Accession number (accession no.) refers to the transcriptome of *Hyas araneus* [40] and the database ENA (EMBL). Details on transcript description and transcript length are listed for each transcript. Transcripts are sorted according to the rank in absolute regulation regardless of the treatment. Changes are given in log_2_-fold change for each treatment separately. Bold numbers represent significantly up-regulated transcripts; bold and italic numbers represent significantly down-regulated transcripts.

#### Energy metabolism

Acid–base regulation compensating for CO_2_ induced disturbances likely entails a metabolic cost due to the ATP demanding movement of H^+^ by ion transporters [25]. Elevated metabolic rates have already been observed under hypercapnia exposure [26, 63], however, the mechanisms causing such cost increments remain unidentified. The differential expression analysis revealed an up-regulation of complex I and complex IV of the electron transport system (ETS) during exposure to intermediate CO_2_ levels (i,iv) (Table 2). Transcripts encoding for NADH dehydrogenase and cytochrome c oxidase subunits were significantly up-regulated above control levels possibly leading to an increase in mitochondrial density to meet the increased energy demand. Interestingly, complex II and III were not affected by hypercapnia exposure. Increased activities of complex I and IV activated by protein kinase A (PKA) resulted in an increased oxidative phosphorylation and ATP synthesis in human kidney cells [64]. The activation was triggered by a soluble adenylyl cyclase (sAC) induced signalling pathway that implies phosphorylation of PKA being stimulated by cyclic adenosine monophosphate (cAMP), which, in turn, is formed from sAC. In mammals and in elasmobranchs it is known that sAC is stimulated by HCO_3_^−^ [65, 66], which would thereby support an increased activity of mitochondrial electron transport system under hypercapnia exposure. cAMP and PKA regulate key enzymes, such as complex IV, by alterating gene expression [66, 67]. Considering the higher extracellular bicarbonate levels in animals under hypercapnia (Figure 4) this signalling pathway possibly led to the increased up-regulation in ETS related genes and/or increased activity and thus might explain how the organisms meet the suggested increase in ATP demand (Figure 4).

An increased ATP production by the ETS would also lead to an elevated demand for metabolic substrates and turnover of the resulting reduction equivalents (NADH, FADH_2_). In the intermediate CO_2_ treatments (I, IV), the glycolytic pathway only experienced a significant up-regulation of glyceraldehyde-3-phosphate dehydrogenase (GAPDH) (Table 2), whereas the rate-limiting enzymes phosphofructokinase or pyruvate kinase remained unaffected, suggesting no general up-regulation of glycolysis. However, the enhanced expression of sodium glucose transporter indicates increased capacity for glucose transport from the hemolymph into the gill cells (Table 2). Trehalose is the major hemolymph sugar in insects and decapod crustaceans, with higher levels than glucose [68, 69]. Enzymes for trehalose synthesis were found in crustacean tissues, including gills [70]. Trehalose and its fast transport into cells and consecutive transformation into glucose reflect its immediate availability to meet sudden bouts of energy demand. Accordingly, a 6.7 fold increase of trehalose concentration was measured in the hemolymph of *C. maenas* over 10 days under osmotic stress [71]. In *H. araneus,* however, the expression of trehalose-6-phosphate synthase was significantly down-regulated in all treatments (I-V) suggesting suppressed synthesis of the already depleted trehalose stores (Table 2). Significant up-regulation of transcripts encoding for glycogen phosphorylase (I, IV) and alpha glucosidase (IV) was found instead. Both enzymes catalyse the glucose releasing steps of glycogenolysis indicating the use of glycogen as a glucose source during long-term increased demand.

Enhanced demand for glucose is paralleled by a down-regulation of glucose-6-phosphate dehydrogenase (G6PD) in all treatments (I-V) indicating the potentially reduced production of NADPH (Table 2). G6PD is the key enzyme of the oxidative phase of the pentose phosphate pathway, the main source of NADPH for biosynthetic pathways in the cells (Figure 4). Furthermore, the cytosolic (NADP dependent) isocitrate dehydrogenase (IDH) was significantly down-regulated under intermediate CO_2_ levels at high temperature (IV), also suggesting lowered biosynthetic rates such as lipid biosynthesis under combined exposure (Table 2).

#### Oxidative stress

Besides being involved in lipid biosynthesis, NADPH is an important reducing agent in cellular antioxidative defence, e.g. by regenerating reduced glutathione, a major cellular antioxidant (Figure 4). Thus, besides a general down-regulation of anabolic reactions it seems conceivable that *H. araneus* encounters a reduced capacity to counteract oxidative stress under hypercapnic and thermal stress.

Significant changes in the expression of several genes involved in cellular antioxidant defence, including several peroxidases, indicate potential oxidative stress in *H. araneus* under intermediate CO_2_ exposure (I). These changes in expression level were less pronounced under high CO_2_ (II), suggesting a decreasing acclimation capacity of the *H. araneus* with increasing external *P*CO_2_(Table 2, see above). Among up-regulated genes, especially genes associated with the detoxification of hydrogen peroxide (H_2_O_2_) were affected under hypercapnia exposure. An ascorbate peroxidase (APX) was significantly up-regulated under intermediate CO_2_ concentrations (I). APX is a peroxidase that utilizes ascorbate as electron donor to detoxify H_2_O_2_ into water (Table 2). Additionally, a glutathione peroxidase (GPX) that reduces H_2_O_2_, using glutathione as substrate, was up-regulated under high CO_2_ exposure (II). This contrasts the down-regulation of two thiol-specific peroxiredoxin-1, at intermediate CO_2_ concentration (I), indicating a balanced response possibly by differential transcription of different splice variants. Peroxiredoxins are ubiquitous enzymes detoxifying peroxides, such as H_2_O_2_, by oxidising their active cysteine site using peroxide as substrate and are regenerated by oxidation of a thiol-containing electron donor, commonly thioredoxin [72]. However, only two sequences encoding for a thiol-containing protein (thioredoxin-1) were significantly down-regulated (II, IV).

The up-regulation of genes for anti-oxidants, such as glutathione peroxidase and peroxiredoxin, may indicate compensation for enhanced ROS (reactive oxygen species) production and concomitantly oxidative stress in the gill tissue of *H. araneus* under CO_2_ exposure. This is further supported by a significant up-regulation of a ribosomal cytochrome p450 like gene in the intermediate CO_2_ treatment (I). Cytochrome p450 is involved in the oxidative metabolism of a variety of organic substrates and incomplete catalytic processes can result in a continuous release of ROS [73, 74]. In contrast, a transcript encoding for urate oxidase (uricase) was significantly down-regulated in all treatments (I-V) (Table 2). Uricase is commonly located in the peroxisomes of the hepatopancreas tissue, however, uricase activity has also been detected in gill tissue of the kuruma shrimp *Marsupenaeus japonicas* [75]. Uricase catalyses the reaction from urate to allantoin and contributes to the generation of H_2_O_2_ by the oxidation of uric acid [76]. Consequently, a down-regulation of uricase may contribute to alleviate the generation of ROS. Interestingly, CO_2_ exposure also led to an increase in several vitellogenin like transcripts (Table 2). Although expression of vitellogenins is generally sex- and tissue-specific, the expression in both sexes of the mud shrimp *Upogebia major* [77], revealed a positive effect on oxidative stress resistance regardless of the developmental stage. Vitellogenin is also beneficial for oxidative stress resistance in honeybees, *Apis mellifera* [78]. Even if the function of vitellogenin in oxidative stress resistance is far from being completely understood and further investigations are needed to validate this hypothesis, the strong up-regulation of vitellogenin under CO_2_ (I, II, IV) may indicate that vitellogenin is an important protein in the resistance to CO_2_-induced oxidative stress (see below).

Hypercapnia induced enhancement of oxidative stress defence was recently demonstrated in the Eastern oyster, *Crassostrea virginica* [79]. In a proteomic approach, an up-regulation of several proteins, e.g. superoxide dismutase and several peroxiredoxins, was detected after exposure to high *P*CO_2_ (~3,520 *μ*atm) for 2 weeks. The authors suggested several ways of how increased CO_2_ levels could directly or indirectly cause oxidative stress. On the one hand, a reaction of CO_2_ with peroxynitrite, a ROS formed through the reaction between superoxide anions and nitric oxide, resulting in the formation of reactive carbonate and nitrogen species, can lead to oxidative stress by oxidizing molecular compounds [80]. On the other hand, an indirect influence of elevated CO_2_ and/or pH could adversely affect mitochondrial functions and/or the non-enzymatic production of ROS [79]. However, our findings cannot support a direct influence of CO_2_ on oxidative stress generation, as one would assume an increase in the response to oxidative stress with increasing seawater CO_2_. Our data rather indicate an indirect influence of elevated CO_2_ on ROS production, likely by enhanced ROS production due to metabolic stimulation. The suggested increase in oxidative metabolic processes might cause enhanced ROS production and would also explain why the oxidative stress response was higher under intermediate CO_2_ than in the high CO_2_ treatment.

#### Cell structure

It is well known that the formation of ROS can damage lipids, proteins and DNA e.g. [81]. A large group of genes that belong to the functional category *‘cytoskeleton’* include several actins and tubulins which are up-regulated together with the antioxidant genes under CO_2_ exposure. The cytoskeleton is one major target for oxidative stress (Table 2) when the exposed cysteine component of actin forms oxidized derivates, such as intermolecular disulfide bridges [82]. This presumably has adverse effects on the interaction between actin and actin binding proteins and leads to changes in the structure of the actin cytoskeleton. The up-regulation of two transcripts encoding for actin in both intermediate CO_2_ treatments (I, IV) and the high CO_2_ treatment (II) may counter the damages caused by oxidative stress. An additional up-regulation of two actin binding proteins, nesprin and adducin, further supports the need for structural adaptation under oxidative stress triggered by CO_2_ exposure (Table 2). There is strong evidence that ROS induce the expression of tubulin [83], which could explain the up-regulation of *β*-tubulin and three transcript sequences encoding forα-tubulin in both intermediate CO_2_ treatments (I, IV) and the high CO_2_ (II) treatment (Table 2). Although the effect of oxidative stress on the cytoskeleton is still poorly understood and needs further investigation, an interaction of oxidative stress and adaptive changes in the cytoskeleton is well recognized [79, 82] and supported by our findings.

Besides enhanced antioxidative defence, a reorganisation of the cytoskeleton may occur in gill epithelia during the initial stage of hypercapnia acclimation. In *C. maenas*, a reorganisation of gill epithelia was observed after short-term exposure to very high *P*CO_2_ (~4,340 *μ*atm; 7 days), but not after medium-term exposure to high *P*CO_2_ (~2,270 *μ*atm; 11 weeks) [36]. Similarly, a reorganization of the cytoskeleton may not occur during medium-term CO_2_ acclimation of *H. araneus* (10 weeks). The response of genes that belong to the functional category *‘cytoskeleton’* was higher at intermediate CO_2_ (I, IV) than at high CO_2_ (II), in line with the findings in antioxidative defence. This also suggests that the changes observed result from oxidative stress via enhanced metabolic rate at intermediate CO_2_ with the cytoskeleton as a direct target of ROS formation.

Cytoskeletal genes also responded to elevated temperature (III). Several genes encoding for actin-binding proteins as well as membrane and cuticle proteins were significantly up-regulated (Table 2). Three transcripts of gelsolin, an actin-binding protein and regulator of actin filament assembly, were significantly up-regulated. The expression of an actin-binding nesprin, a membrane-binding ankyrin and an actin-related cytoskeletal structure protein spectrin was also increased. Warming affects various cellular processes, including stability and/or dynamics of the cytoskeleton for review see [84]. An increase in cytoskeletal gene expression despite the general down-regulation of expression levels indicates a requirement for the stabilization of the cytoskeleton, possibly elicited by the warm-induced stimulation of metabolic rate and associated ROS formation. Interestingly, such up-regulation of cytoskeletal gene expression could not be seen in under combined intermediate *P*CO_2_ and high temperature (IV, V), suggesting that the general down-regulation induced by high CO_2_ also affects cytoskeleton assembly.

Commonly, stresses such as temperature extremes, cellular energy depletion, and extreme concentrations of ions, osmolytes and gases induce the synthesis of heat-shock proteins (HSP) for review see [85]. However, HSP expression levels being unchanged in the high temperature treatment (III) suggest that 10°C is not a temperature extreme for the Arctic *H. araneus* population. In contrast, the expression of several HSPs was up-regulated under CO_2_ exposure (Table 2). One HSP70 was up-regulated in both the intermediate and the high CO_2_ treatments (I, II, IV). Four transcripts encoding for HSP90 were significantly up-regulated in the intermediate CO_2_ treatment (I) and two in the intermediate CO_2_ plus high temperature treatment (IV). In treatments I, III, and V one HSP90 showed decreased expression. Acting as molecular chaperones, HSPs can play an important role in maintaining proteins in a folded or unfolded state, controlling the unintended aggregation of proteins or target proteins for degradation [86]. Their induced expression, especially under intermediate CO_2_ concentrations, paired with increases in mRNA transcripts for proteins involved in antioxidant defence suggest an increased capacity to defend the cell against cellular damage. The up-regulation specifically of HSP90 suggests HSP90 to be of particular importance in maintaining protein homeostasis.

## Conclusions

**Figure 5:**
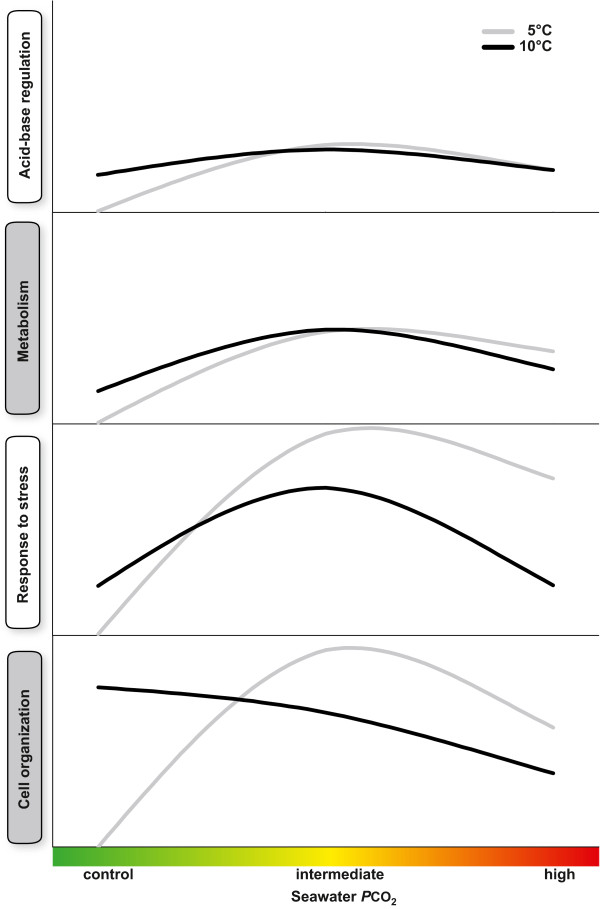
**Schematic description of the response of** ***Hyas araneus*** **to different exposure experiments for specific categories.** For each treatment, log_2_-fold changes of all up-regulated transcripts within one specific category were summed and plotted as a line (gray line = 5°C; black line = 10°C). Scales are harmonized for all categories. The figure shows how acclimation to warming reduces the transcriptomic response of ‘cell organization’ and ‘response to stress’ to elevated CO_2_ tensions.

Based on a comprehensive expression analysis of genes related to acid–base regulation, metabolism, cell structure and their coupling to the stress response, this study has identified moderate, but distinct responses to ocean acidification in gills of adult *H. araneus*. We could demonstrate that the molecular response strongly depends on the CO_2_ concentration. At *P*CO_2_ values proposed for the end of the century, changes in expression suggest elevated metabolism caused by stimulated acid–base regulation and associated with increased oxidative stress. This up-regulation was attenuated at even higher *P*CO_2_ (business as usual scenario for Year 2300) with expression levels closer to control values (Figure 5), indicating down-regulation of these processes. Responses in transcripts related to acid–base regulation and energy metabolism were in line with those observed at elevated temperature. Observations under hypercapnia in the blue mussel *M. edulis* are in line with these conclusions. An increase in aerobic metabolism under intermediate *P*CO_2_ exposure (<2,400 *μ*atm) was followed by a decrease at higher *P*CO_2_ [87, 88]. In earlier studies on marine invertebrates, high *P*CO_2_ exposure (~3,200-5,000 *μ*atm) induced a decrease in metabolic rates. In the mussel *Mytilus galloprovincialis* and the decapod crab *Necora puber*, a decrease in respiration rates was seen in response to long-term exposure at a *P*CO_2_ of approx. 5,000 and 3,200 *μ*atm, respectively [6, 9]. Our findings suggest a *P*CO_2_ dependent threshold where metabolic stimulation might turn into metabolic depression. In *H. araneus* this threshold may be reached at a *P*CO_2_ of approximately 2,000 *μ*atm. Although further examinations besides transcriptomics covering a broader range of *P*CO_2_ concentrations are certainly needed to confirm a possible threshold, a recent meta-analysis revealed that 50% of all marine crustacean species were negatively affected at this *P*CO_2_ [5].

Changes in the expression of genes related to energy metabolism paralleled the expression of those related to acid–base regulation and both paralleled the changes in extracellular pH (pH_e_), indicating a feedback regulation between pH and metabolic rate (Figure 5). In the marine worm *S. nudus* and the mussel *M. galloprovincialis*, metabolic depression under CO_2_ was caused by a drop in pH_e_ [6, 27, 89]. *H. araneus* was able to partially compensate for the acidosis via bicarbonate (HCO_3_^−^) accumulation, but capacities were not sufficient to fully compensate. At both experimental temperatures (5°C and 10°C) hemolymph acidosis was compensated to the same extent. At high CO_2_ concentrations, the capacity to fully compensate may be even more limited related to decreased ion transporter expression and activities. Since acid–base regulation is an ATP-consuming process, the decreased expression of metabolism related genes might at least in part be explained by a pH_e_-triggered inhibition.

These shifts cause changes in energy balance which can therefore reflect the limits in stress tolerance [90]. A bioenergetic framework [90] builds on the physiological concepts of oxygen- and capacity-limited thermal tolerance (OCLTT) [91–93] and the dynamic energy budget (DEB) [94]. Two cases distinguished between physiological responses to moderate and extreme environmental stress. Moderate stress induces additional ATP turnover along with a higher metabolic rate, in *H. araneus* exemplified in enhanced acid–base regulation and cellular damage repair from oxidative stress at intermediate *P*CO_2_. These mechanisms support long-term persistence at the expense of a shift in energy budget (*pejus* range) [95]. Even if the enhanced energy demand of stress resistance can be met by increased feeding, it may still result in a reallocation of energy from fitness-related functions such as reproduction and growth to maintenance and damage repair [90]. More extreme stress exacerbates the disturbances in homeostasis, exemplified in stronger disturbances in acid–base status as seen in *H. araneus* favouring a suppression of metabolic rate [27] which preserve energy resources and lessen the generation of detrimental metabolic by-products [90]. In *H. araneus* the respective response to high *P*CO_2_ was paralleled by a lower expression of transcripts associated with energy metabolism, stress response and cellular organization. Depressed metabolism would result in insufficient energy balance and time-limited tolerance to environmental stress making long-term survival impossible (*pessimum* range) [90, 93].

To our knowledge, we report the first gene expression profiling study analysing the responses to a combination of two drivers in an osmoconforming crustacean. The study confirms the interdependence of physiological processes affected by elevated seawater *P*CO_2_ and temperature. Furthermore, the study demonstrates the importance of considering projected climate scenarios in experimental work, as responses to increasing seawater CO_2_ concentrations are not necessarily linear, as presented here.

## Electronic supplementary material

Additional file 1: Table S1: Summary of sequencing and mapping results for *Hyas araneus*. Details on treatment, filename and location of sequencing raw data, used sequencer, read count, read length and total size in base pairs (bp), used alignment tool, aligned reads and percentage of aligned reads are listed separately. (XLSX 57 KB)

Additional file 2: Table S2: Details on primers for quantitative real-time polymerase chain reaction (qRT-PCR) to validate RNASeq data of *Hyas araneus*. Forward and backward primer sequences and descriptions of target genes used in the qRT-PCR. Accession number (accession no.) refer to the transcriptome of *Hyas araneus* [40] and the database ENA (EMBL). R^2^ and efficiency was tested in a qRT-PCR dilution series (for details, see Methods). (XLSX 40 KB)

Additional file 3: Figure S1: Changes of expression levels of transcripts in gills of *Hyas araneus* responding to medium-term exposure (10 weeks) at intermediate *P*CO_2_ (≈1,000 *μ*atm) and high temperature (10°C), analysed by DESeq (gray bars) and quantitative real-time polymerase chain reaction (qRT-PCR) (black bars). Bars represent the mean log_2_-fold change and standard error (error bars) of the respective gene. Transcripts correspond to primers and genes used in the qRT-PCR (see Additional file 5: Table S3). (PDF 78 KB)

Additional file 4: Figure S2: Linear regression between expression levels of transcripts in gills of *Hyas araneus* responding to medium-term exposure (10 weeks) at intermediate *P*CO_2_ (≈1,000 *μ*atm) and high temperature (10°C), analysed by DESeq and quantitative real-time polymerase chain reaction (qRT-PCR). Black dots represent the mean log_2_-fold change of transcripts analysed by DESeq plotted against the corresponding mean log_2_-fold change analysed by qRT-PCR. *r* was determined by Pearson Correlation using SigmaPlot 12.0 (Systat Software Inc., San Jose, USA). (PDF 53 KB)

Additional file 5: Table S3: Transcript levels changing significantly in gills of *Hyas araneus* responding to hypercapnia and elevated temperature. Transcripts regulated significantly in response to hypercapnia and elevated temperature as identified by DESeq analysis (for details, see Methods). ID and accession number (accession no.) refer to the transcriptome of *Hyas araneus* [40] and the database ENA (EMBL). Details on transcript description and transcript length are listed for each transcript. Transcripts are sorted according to the rank in absolute regulation regardless of the treatment. Changes are given in log_2_-fold change for each treatment separately. For details on treatments, see Methods). Bold numbers represent significantly up-regulated transcripts and bold and underlined numbers significantly down-regulated transcripts. (XLSX 100 KB)

Additional file 6: Figure S3: Smearplot of differentially expressed transcripts in gills of *Hyas araneus*. All transcripts changed in response to hypercapnia and elevated temperature. Log_2_-fold changes are plotted against mean readcount (log_10_). Blue dots represent transcripts with non-significant changes, red dots depict transcripts significantly regulated as identified by DESeq analysis (*p* < 0.05) and green triangles are transcripts changed significantly and identified by annotation Numbers refer to the total number of significantly up-/down-regulated transcripts. A) treatment (I) = 1,120 *μ*atm *P*CO_2_ 5°C; B) treatment (II) = 1,960 *μ*atm *P*CO_2_ 5°C; C) treatment (III) = 390 *μ*atm *P*CO_2_ 10°C; D) treatment (IV) = 1,120 *μ*atm *P*CO_2_ 10°C; E) treatment (V) = 1,960 *μ*atm *P*CO_2_ 10°C. (PDF 492 KB)

Additional file 7: Table S4: Enrichment analysis in the RNASeq study on *Hyas araneus*. Results of the enrichment analysis (Fisher’s Exact Test; FDR < 0.05) as implemented in Blast2GO [49, 50] and reduced by web-based clustering tool REVIGO [51]. Tested were subsets of all significantly regulated transcripts as identified by DESeq (separated by up- and down-regulated transcripts). Reference-set was the full set of annotated sequences of the *H. araneus* transcriptome [40]. Listed is the Gene Ontology term (GO-term), the name of the functional group (description), the category (molecular function, biological process or cellular component), the false discovery rate (FDR) and whether GO-terms are over- or under-represented (a GO-term is considered over-/under-represented if it appears significantly more often/less often in the test-set than in the reference-set. (XLSX 33 KB)
