# Supplementary figures and images for: Gene expression profiling in gills of the great spider crab Hyas araneus in response to ocean acidification and warming
Source: BMC Genomics. 2014 Sep 12;15(1):789. doi: 10.1186/1471-2164-15-789 (PMC4176836; doi:10.1186/1471-2164-15-789)

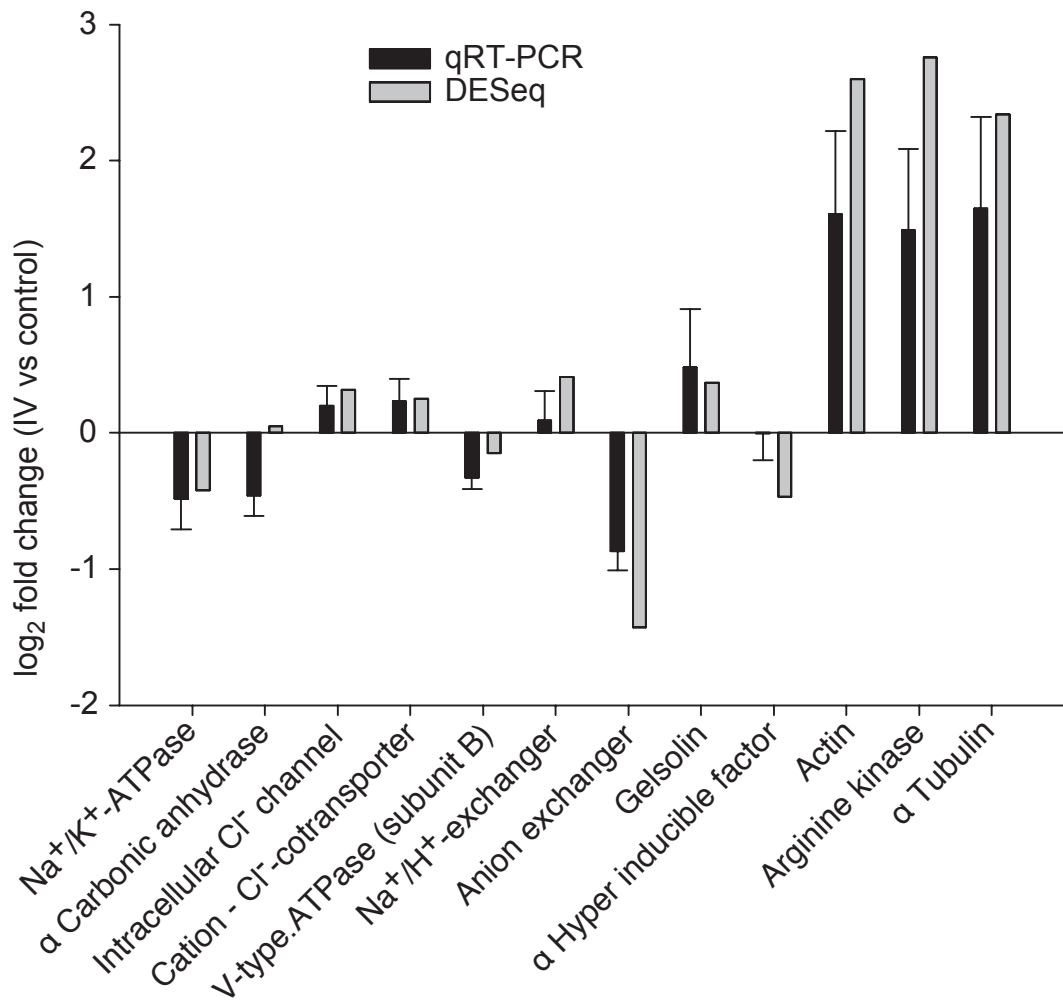

Supplement: Supplementary file 3 — Additional file 3: Figure S1: Changes of expression levels of transcripts in gills of Hyas araneus responding to medium-term exposure (10 weeks) at intermediate PCO2 (≈1,000 μatm) and high temperature (10°C), analysed by DESeq (gray bars) and quantitative real-time polymerase chain reaction (qRT-PCR) (black bars). Bars represent the mean log2-fold change and standard error (error bars) of the respective gene. Transcripts correspond to primers and genes used in the qRT-PCR (see Additional file 5: Table S3). (PDF 78 KB) [file 12864_2014_6468_MOESM3_ESM.pdf]

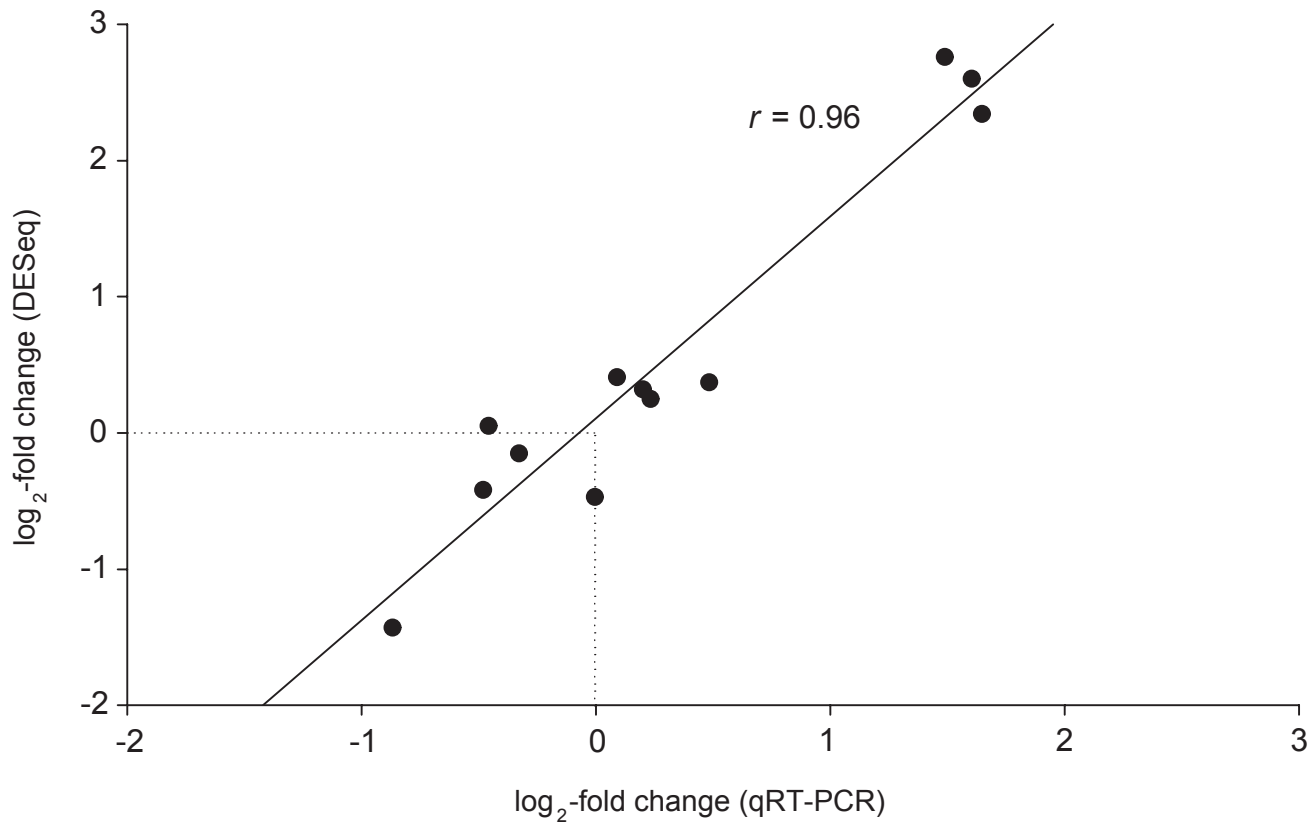

Supplement: Supplementary file 4 — Additional file 4: Figure S2: Linear regression between expression levels of transcripts in gills of Hyas araneus responding to medium-term exposure (10 weeks) at intermediate PCO2 (≈1,000 μatm) and high temperature (10°C), analysed by DESeq and quantitative real-time polymerase chain reaction (qRT-PCR). Black dots represent the mean log2-fold change of transcripts analysed by DESeq plotted against the corresponding mean log2-fold change analysed by qRT-PCR. r was determined by Pearson Correlation using SigmaPlot 12.0 (Systat Software Inc., San Jose, USA). (PDF 53 KB) [file 12864_2014_6468_MOESM4_ESM.pdf]

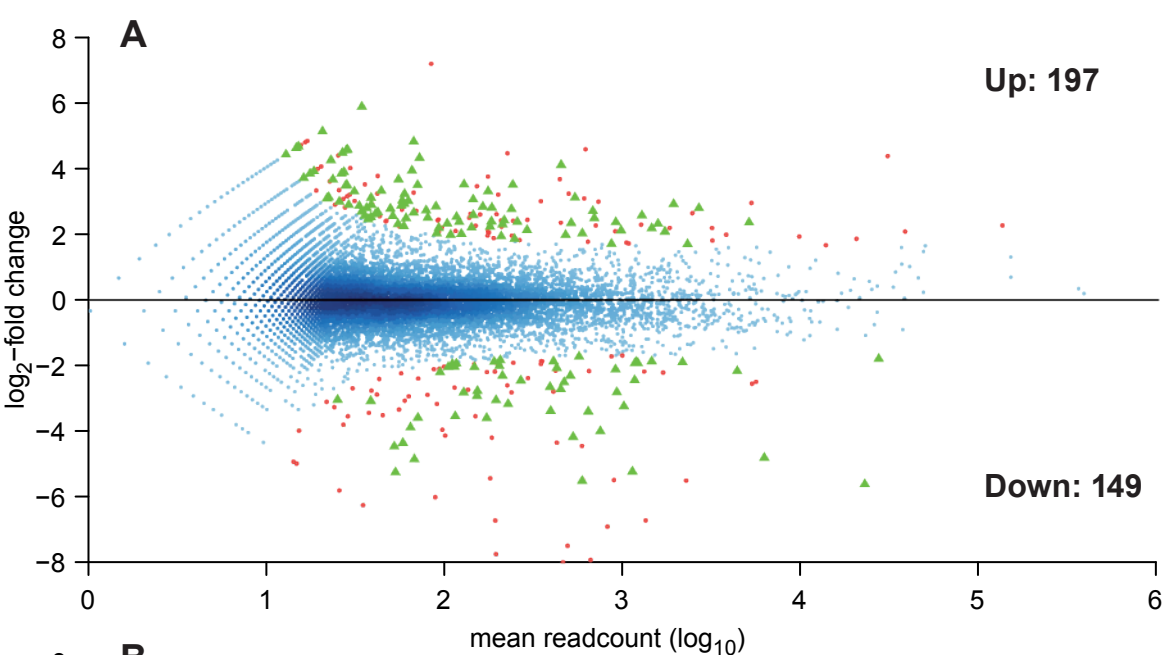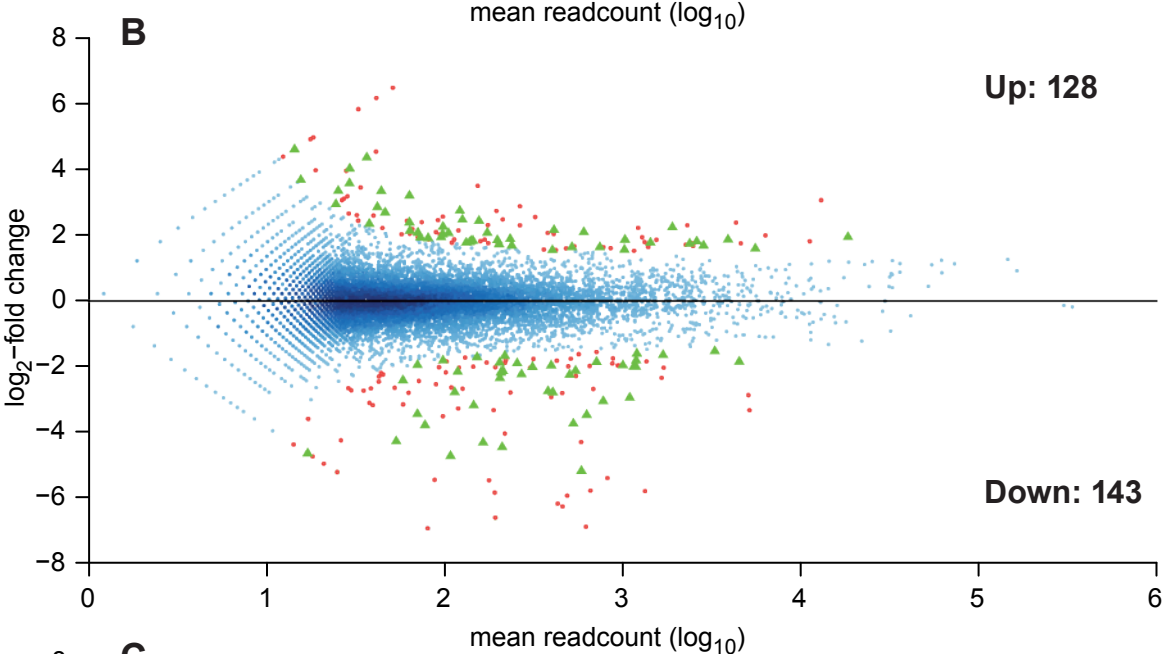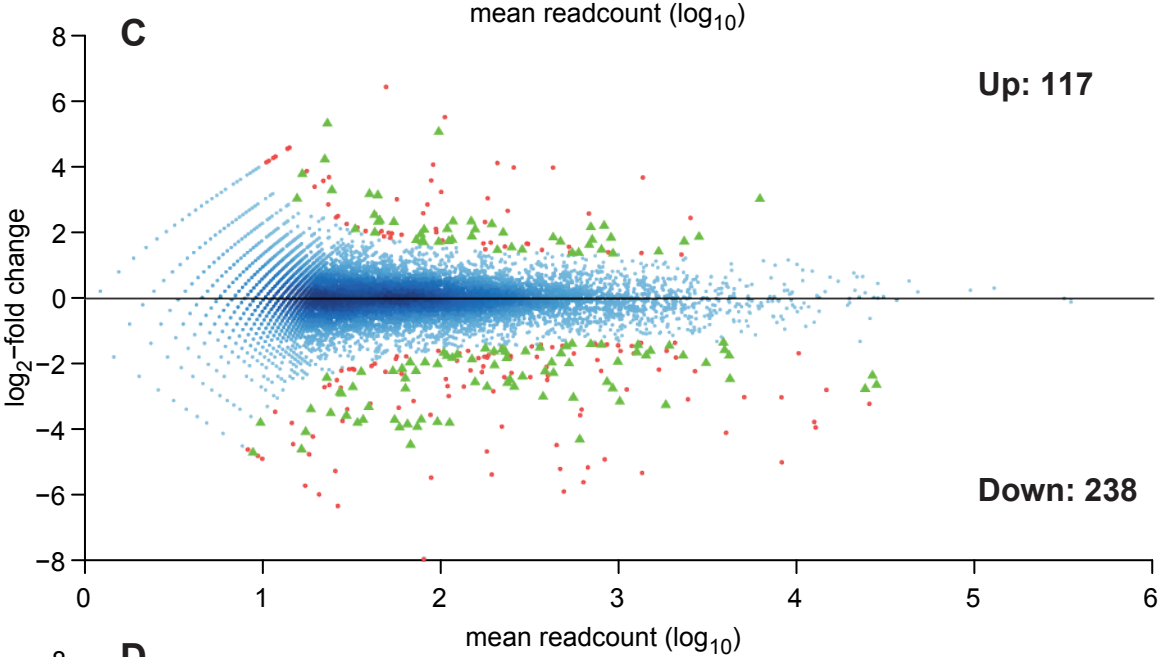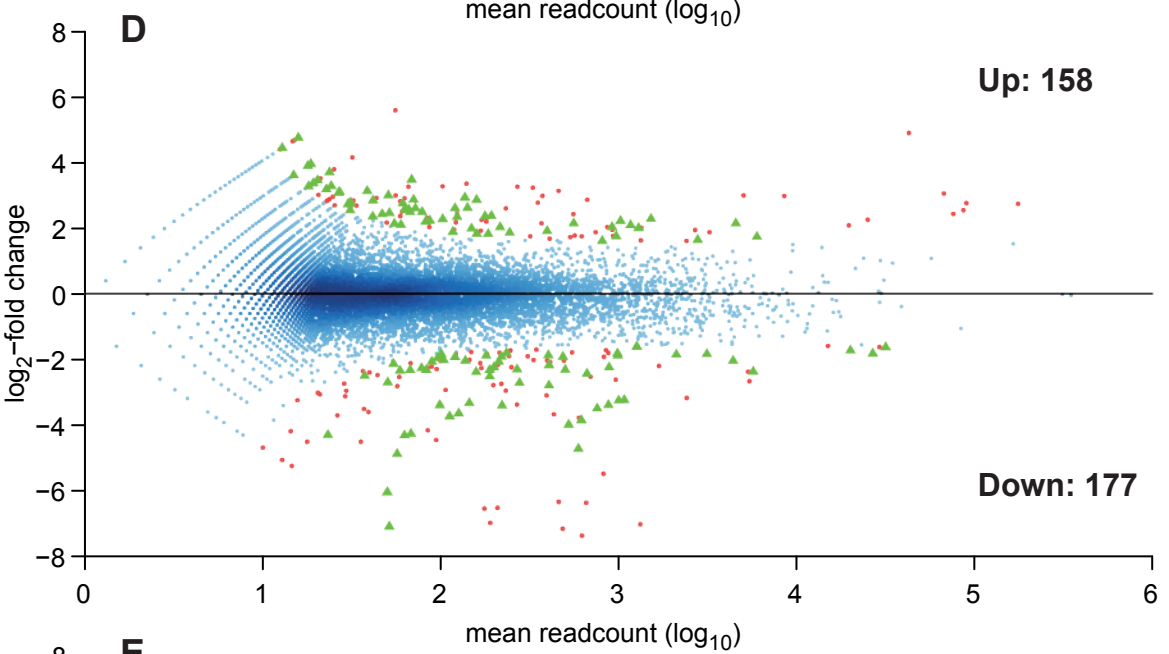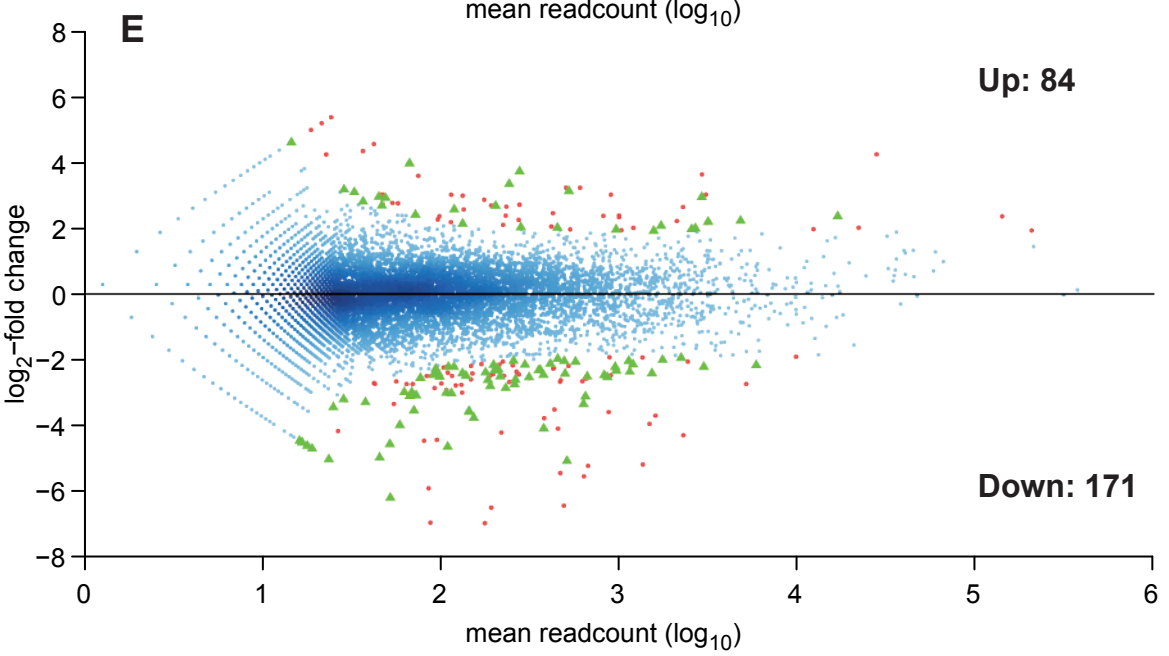

Supplement: Supplementary file 6 — Additional file 6: Figure S3: Smearplot of differentially expressed transcripts in gills of Hyas araneus. All transcripts changed in response to hypercapnia and elevated temperature. Log2-fold changes are plotted against mean readcount (log10). Blue dots represent transcripts with non-significant changes, red dots depict transcripts significantly regulated as identified by DESeq analysis (p < 0.05) and green triangles are transcripts changed significantly and identified by annotation Numbers refer to the total number of significantly up-/down-regulated transcripts. A) treatment (I) = 1,120 μatm PCO2 5°C; B) treatment (II) = 1,960 μatm PCO2 5°C; C) treatment (III) = 390 μatm PCO2 10°C; D) treatment (IV) = 1,120 μatm PCO2 10°C; E) treatment (V) = 1,960 μatm PCO2 10°C. (PDF 492 KB) [file 12864_2014_6468_MOESM6_ESM.pdf]
